# Use of Laser in Periodontal Tissue Regeneration: A Scoping Review of Clinical and Experimental Evidence

**DOI:** 10.3390/medicina61122199

**Published:** 2025-12-12

**Authors:** Martina Bosisio, Umberto Romeo, Alessandro Del Vecchio, Aldo Bruno Giannì

**Affiliations:** 1 Department of Biomedical, Surgical and Dental Sciences, University of Milan, 20122 Milan, Italy; aldo.gianni@unimi.it; 2 Maxillo-Facial Surgery and Dental Unit, Fondazione IRCCS Cà Granda Ospedale Maggiore Policlinico, 20122 Milan, Italy; 3 Department of Oral Sciences and Maxillofacial Surgery, Sapienza University of Rome, 00161 Rome, Italy; umberto.romeo@uniroma1.it (U.R.); alessandro.delvecchio@uniroma1.it (A.D.V.)

**Keywords:** photobiomodulation, laser surgery, periodontal regeneration, tissue regeneration, periodontal ligament, laser regeneration, oral surgery, oral pathology

## Abstract

*Background and Objectives*: Periodontitis leads to progressive destruction of periodontal tissues and, despite advances in regenerative approaches, clinical outcomes remain inconsistent. Lasers have been proposed as adjuncts in regenerative periodontology because of their antimicrobial, hemostatic, and photobiomodulatory properties. However, available evidence remains heterogeneous. This scoping review aims to systematically map clinical and experimental evidence on the role of lasers in periodontal tissue regeneration. *Materials and Methods*: The review was conducted in accordance with the PRISMA-ScR guidelines. PubMed, Scopus, and Web of Science were searched up to September 2025 without time restrictions. Eligible studies included in vitro, ex vivo, in vivo and clinical research assessing the application of lasers for periodontal healing. Reviews, conference abstracts and studies unrelated to regeneration were excluded. *Results*: The electronic search retrieved 314 records, of which 193 unique articles were screened after duplicates removal and 17 full texts were assessed. A total of 15 studies met the eligibility criteria and were included in the review. Included studies comprised 5 in vitro investigations, 2 ex vivo studies, 1 in vivo animal study, 4 case reports and 3 RCTs, published between 2015 and 2025. In vitro and ex vivo evidence demonstrated that laser irradiation enhanced cell proliferation, differentiation, growth factor release, and root surface conditioning. The in vivo study confirmed increased angiogenesis and bone formation after Er:YAG PBM. Clinical studies, including RCTs and case reports, reported improvements in PD reduction, clinical attachment gain, and radiographic bone fill, particularly when lasers were applied as adjuncts to regenerative techniques or biomaterials. *Conclusions*: Available evidence suggests that lasers can positively modulate biological processes and enhance the outcomes of regenerative periodontal procedures. However, the limited number of high-quality clinical trials, variability in laser types and parameters, and heterogeneity in protocols limit the strength of current conclusions. Further standardized RCTs with long-term follow-up are needed to clarify the clinical relevance of lasers in periodontal regenerative outcomes.

## 1. Introduction

Periodontitis is one of the leading causes of tooth loss in adults and is characterized by chronic inflammatory processes that progressively destroy the supporting tissues of the teeth, including periodontal ligament, cementum, and alveolar bone [1].

In recent decades, several strategies have been proposed to promote periodontal regeneration, including regenerative surgical approaches, biomaterials, and adjunctive therapies aimed at enhancing wound healing [2]. However, clinical outcomes remain heterogeneous and are influenced by multiple factors, highlighting the need for auxiliary methods that can improve regeneration [3].

Lasers have been introduced in periodontology as tools combining antimicrobial, hemostatic, and biostimulatory effects, with applications in both non-surgical and surgical periodontal therapy [4]. The photothermal and photobiomodulatory actions of lasers extend beyond root surface decontamination and are able to modulate cellular responses, promoting proliferation and differentiation of fibroblasts and periodontal stem cells and stimulating neoangiogenesis [5,6].

Several reviews have assessed the efficacy of different laser types. Erbium lasers (Erbium:YAG laser (Er:YAG), Erbium, Chromium:YSGG laser (Er,Cr:YSGG)) have proven effective in removing granulation tissue and smear layer, showing promising outcomes in regenerative surgery [7,8]. Neodymium:YAG (Nd:YAG) lasers demonstrated potential in reducing periodontal pathogens and favoring wound healing [9]. Diode lasers and low-level laser therapy (LLLT)/photobiomodulation (PBM) have been associated with improved cellular responses and enhanced recovery of clinical periodontal parameters [5,10].

Nevertheless, available scientific evidence is not always consistent. Some studies reported significant improvements in probing depth (PD) reduction and clinical attachment level (CAL) gain, while others found no relevant differences compared to conventional therapy [1,3]. These discrepancies are mainly attributed to heterogeneity in clinical protocols, irradiation parameters, experimental models, and laser types employed [11,12].

Alongside clinical investigations, basic research has provided insights into the biological mechanisms underlying laser applications in regenerative periodontology. Specific wavelengths have been shown to activate intracellular signaling pathways such as Mitogen-Activated Protein Kinase (MAPK)/ Extracellular Signal-Regulated Kinase (ERK) and stimulate the expression of osteogenic factors, thus favoring new periodontal tissue formation [6].

Despite the increasing interest, there is still a lack of comprehensive syntheses that systematically map the clinical, in vivo and in vitro evidence on laser applications in regenerative periodontal processes. Previous reviews have mainly focused on single laser types or specific therapeutic approaches [3,7,9], leaving a gap for an overarching evaluation of the available evidence.

Therefore, the aim of the present scoping review is to provide a comprehensive and up-to-date overview of clinical and experimental evidence on the use of lasers in periodontal tissue regeneration, identifying the laser types studied, irradiation parameters, experimental models, and the major gaps in the current literature.

## 2. Materials and Methods

### 2.1. Protocol

This scoping review adhered to the guidelines outlined in the Preferred Reporting Items for Systematic Reviews and Meta-Analyses extension for Scoping Reviews (PRISMA-ScR) to comprehensively synthesize existing evidence and identify key concepts regarding the application of lasers in periodontal tissue healing (Table S1, Supplementary Materials) [13].

An adapted version of the PICO (Population, Intervention, Comparison, and Outcome) model was employed to formulate a focused question structured around a PEO (Population, Exposure, and Outcome) framework. This approach was utilized to evaluate the relationship between the exposure to laser therapy and the resulting biological and clinical outcomes in periodontal regeneration. It has previously been adopted in qualitative systematic reviews of healthcare interventions, including procedures in oral and periodontal surgery [14,15].

The main question of this scoping review was “Does the use of laser therapy improve the potential for periodontal tissue regeneration in clinical and in experimental models?”

To address this question, studies reporting outcomes of periodontal regeneration following the application of lasers were analyzed in order to clarify the impact of different wavelengths and protocols on periodontal healing.

This scoping review was preregistered on Preprints.org (DOI: https://doi.org/10.20944/preprints202511.0169.v2).

### 2.2. Eligibility Criteria

#### 2.2.1. Inclusion Criteria

All sources of evidence had to satisfy specific inclusion criteria to be considered. These encompassed articles written exclusively in English, with no restrictions based on publication date. Eligible study designs included randomized controlled trials (RCTs), prospective and retrospective observational studies, case–control studies and case reports. In addition, experimental studies conducted in vitro, ex vivo, or in vivo (animal models) were incorporated. For clinical evidence, RCTs were considered the highest level of included evidence, whereas case reports and observational studies were included only for descriptive and exploratory purposes.

The primary focus of the investigation in these studies had to be the evaluation of periodontal healing promoted by laser therapy, with particular emphasis on both clinical and experimental research.

#### 2.2.2. Exclusion Criteria

Any studies that did not meet the specified inclusion criteria were excluded from the review. This included articles written in languages other than English, literature reviews (narrative, systematic, or scoping), conference abstracts, and studies that primarily focused on non-regenerative applications of lasers (such as peri-implantitis management, endodontic procedures, tooth whitening, or soft tissue aesthetics).

#### 2.2.3. Search Strategies and Information Source

To perform this review, the PEO model (Population, Exposure, and Outcome) was adopted for structuring the search strategy across multiple electronic databases, including PubMed, Scopus, and Web of Science. The final search was conducted in September 2025.

The PEO model [15] was based on the elements of Population (no restriction was applied to a specific population, as both human and experimental studies were considered), Exposure (evidence from clinical studies (RCTs and Case reports), in vivo, ex vivo, and in vitro studies related to the use of laser therapy), and Outcome (periodontal tissue regeneration).

For each database, the search strings were constructed by combining controlled vocabulary (MeSH terms in PubMed) and free-text keywords related to periodontal disease, laser therapy and regenerative outcomes using Boolean operators (“AND”, “OR”). The search strategy was developed iteratively by two reviewers (M.B. and U.R.) to maximize sensitivity while maintaining specificity for studies on periodontal regeneration. Articles were selected according to predefined search terms related to lasers, periodontal disease, and regeneration. The complete search strings used in PubMed, Scopus, and Web of Science are reported in Table 1.

#### 2.2.4. Selection of Sources of Evidence

Two independent reviewers (MB and U.R.) carried out the initial screening of titles and abstracts for all retrieved articles. Any duplicate entries across the databases were identified and eliminated using the EndNote Web reference manager software (version 20), by Clarivate Analytics, based in Philadelphia, PA, USA. Full-text articles were then individually assessed for eligibility, and studies that fulfilled the predefined inclusion criteria were selected.

The two reviewers compared their selections, and in the case of discrepancies, these were discussed and resolved by consensus or with the intervention of additional reviewers (A.D.V. and A.B.G.)

#### 2.2.5. Methodological and Reporting Quality Assessment

Since this scoping review aims to map the scientific literature on the role of lasers in regeneration of periodontal tissues by synthesizing all available studies to date, including RCTs, Case Reports, in vivo, ex vivo and in vitro research, in accordance with the PRISMA-ScR guidelines, a formal quality assessment of the included studies was not performed.

#### 2.2.6. Analysis of Included Studies

Following the review of the publications, a spreadsheet was generated and sequentially updated during the data extraction process. Separate tables were created for in vitro (Table 3), ex vivo (Table 4), in vivo (Table 5) and clinical studies which consist of three randomized controlled trials and four case reports (Table 6).

For the in vitro studies, the collected data were organized into tables to provide a structured presentation of information, including the name of the first author and year of publication, the type of cells investigated (e.g., periodontal ligament stem cells (PDLSCs), gingival fibroblasts), the type of laser and irradiation parameters, the experimental groups investigated, the assays performed (e.g., proliferation, differentiation, migration), and the main results.

For the ex vivo studies, the data were organized into tables including the first author and year, the type of biological sample (e.g., platelet concentrates, root surfaces), the type of laser and irradiation parameters, the experimental and control groups, the type of analyses performed (e.g., growth factor release, smear layer removal, fibrin adhesion), and the main findings.

For the in vivo study, the extracted information included the first author and year, the animal model used, the type of study, the type of laser and irradiation parameters, the experimental groups, the follow-up period, histological and molecular analyses (e.g., angiogenesis, Vascular Endothelial Growth Factor (VEGF) expression, new bone formation), and the outcomes.

For the clinical studies, RCTs and Case reports, the data were structured into tables reporting the first author and year, the study design, the patient/sample size, the type of laser and irradiation parameters, the adjunctive biomaterials or techniques employed, the comparator groups, the follow-up period, the outcomes assessed (e.g., PD, CAL, radiographic bone fill, molecular biomarkers) and the key findings.

#### 2.2.7. Methodological Appraisal of Randomized Controlled Trials

A structured methodological appraisal of the included randomized controlled trials was performed to support interpretation of the clinical evidence. In accordance with scoping review methodology, this appraisal followed a qualitative, domain-based approach rather than a formal risk-of-bias tool. The evaluation focused on key aspects of internal validity, including the adequacy and reporting of randomization procedures, the presence or absence of blinding of operators and outcome assessors, the appropriateness of the control groups, the completeness of outcome reporting, and sample size considerations. This approach was intended to contextualize the methodological strengths and limitations of the included trials without generating a quantitative quality score. A summary of the appraisal is presented in the Results section.

## 3. Results

The electronic search retrieved a total of 314 records: 122 from Scopus, 65 from PubMed, and 127 from Web of Science. After removing duplicates, 193 unique articles were screened by title and abstract. Of these, 17 full texts were evaluated for eligibility. Finally, 15 studies were included in the scoping review, while 2 were excluded based on the reason that they addressed esthetic papilla management without evaluating regenerative outcomes in periodontology [16,17].

The review included 15 studies: five in vitro [6,18,19,20,21], two ex vivo [22,23], one in vivo (animal) [24], and seven clinical investigations, which consisted of three randomized controlled trials [25,26,27] and four case reports [28,29,30,31]. The publication period ranged from 2015 to 2025.

**Figure 1:**
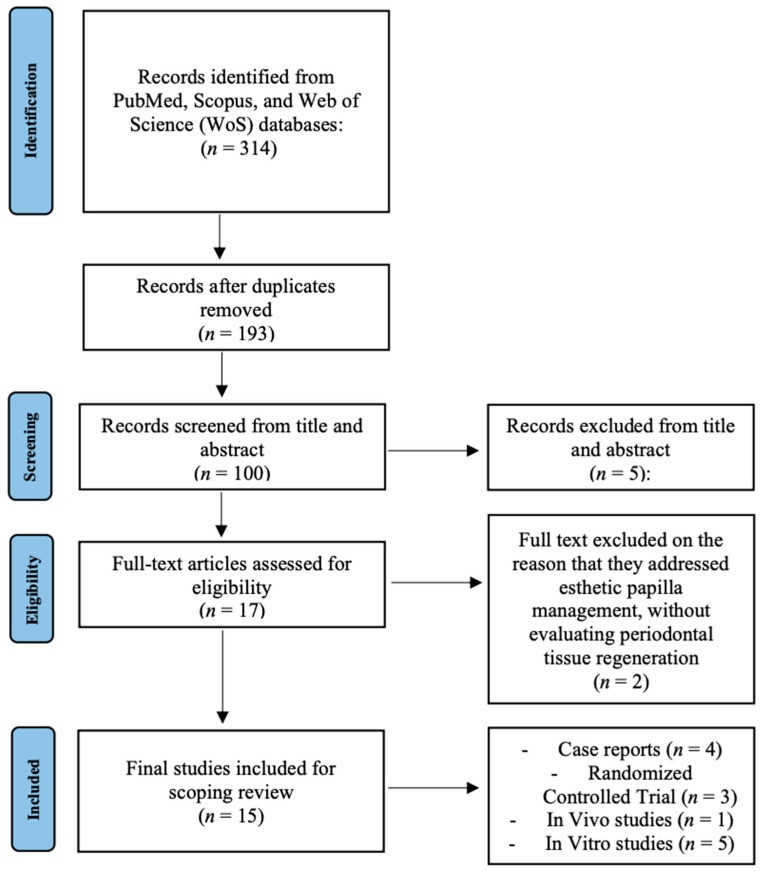
Flowchart of the review process.

The search strategy and study selection process are summarized in Figure 1 (PRISMA-ScR flowchart).

### 3.1. Methodological Appraisal of Randomized Controlled Trials

The methodological appraisal of the three included randomized controlled trials revealed several recurrent limitations affecting their internal validity. Randomization procedures were not always reported in sufficient detail, and two studies did not specify the method used to generate or conceal the allocation sequence. None of the trials implemented blinding of operators or outcome assessors, which increases the risk of performance and detection bias. All RCTs included a control group, although the description of comparator treatments varied in completeness across studies. Outcome reporting was generally adequate, but selective reporting could not be excluded, as some studies did not clearly state whether all predefined outcomes had been presented. All RCTs were conducted with relatively small samples, which may reduce statistical robustness and make true effects more difficult to identify. Collectively, these factors indicate a moderate to high level of methodological limitation across the randomized trials included in this review. The detailed appraisal is presented in Table 2.

### 3.2. Results of Individual Sources of Evidence

The studies included in this scoping review investigated the role of different types of laser devices in periodontal tissue regeneration, using in vitro, ex vivo, and in vivo studies, RCTs and Case Reports.

In vitro studies mainly explored the effects of PBM and laser irradiation on periodontal ligament stem cells, gingival fibroblasts, and osteogenic pathways, consistently demonstrating enhanced cell proliferation, migration, differentiation, and mineralization following irradiation with diode, Nd:YAG, Er:YAG, Er,Cr:YSGG, or Light-Emitting Diode (LED) devices [6,18,19,20,21].

Ex vivo investigations assessed the impact of laser irradiation on platelet concentrates and root surfaces. These studies reported increased release of platelet-derived growth factors from irradiated Platelet-Rich Fibrin (PRF) and Advanced Platelet-Rich Fibrin (A-PRF), and effective removal of smear layer with improved fibrin adhesion on diseased root surfaces, suggesting that lasers may enhance the biological potential of adjunctive regenerative materials [22,23].

The only in vivo study was conducted in a rat model of periodontal defects, confirming that low-level Er:YAG laser irradiation significantly promoted angiogenesis, upregulated VEGF expression, and supported new bone formation, thereby strengthening the translational link between preclinical and clinical applications [24].

Clinical evidence comprised RCTs and Case Reports evaluating the adjunctive use of lasers in regenerative periodontal procedures. Most clinical investigations reported improvements in PD reduction, CAL gain, and radiographic bone fill, particularly when lasers were applied in combination with regenerative approaches such as guided tissue regeneration (GTR), platelet concentrates, or papilla preservation techniques [25,26,27,28,29,30,31]. 

The detailed characteristics and main findings of the included studies are summarized in Table 3, Table 4, Table 5 and Table 6, which present, respectively, the in vitro investigations, the ex vivo studies, the in vivo animal experiment, and the clinical evidence on the use of lasers in periodontal tissue regeneration.

**Table 3:** In vitro studies on laser applications for periodontal regeneration.

Author, Year	Study Design & Sample	Intervention	Comparator	Outcomes	Key Findings
Yamauchi, 2017 [6]	In vitro study on PDLSCs obtained from human third molars, cultured under osteogenic conditions.	Cells irradiated with a high-power red LED at 650 nm, energy density of 8 J/cm^2^, applied in multiple sessions to simulate PBM therapy.	Non-irradiated PDLSCs cultured under the same conditions served as controls.	Assessed proliferation rate, ALP activity, calcium deposition, and ERK1/2 signaling pathway activation.	LED irradiation promoted PDLSC proliferation, significantly increased ALP activity and calcium deposition, and upregulated osteogenic marker expression. Activation of ERK1/2 confirmed a molecular mechanism underlying the observed effects.
El-Dahab, 2024 [18]	In vitro study on human PDLSCs isolated from extracted teeth and cultured under standard conditions.	Cells were irradiated with a diode laser at 970 nm using parameters consistent with PBM protocols. Irradiation was performed at multiple time points to assess cumulative effects on proliferation and differentiation.	Non-irradiated PDLSCs cultured in parallel were used as controls.	Cell proliferation assessed by CCK-8 assay; osteogenic differentiation evaluated via ALP activity, mineralized nodule formation, and gene expression of osteogenic markers.	Laser irradiation significantly enhanced PDLSC proliferation and osteogenic differentiation compared with controls, with greater mineralized nodule deposition, supporting the role of diode lasers in periodontal regeneration.
Aljabri, 2025 [19]	In vitro study using PDLSCs exposed to GMSCs conditioned medium, designed to mimic paracrine signaling in regeneration.	Diode laser at 980 nm applied under LLLT parameters. Irradiation was performed in conjunction with GMSCs conditioned medium to test synergistic effects.	PDLSCs without laser and without conditioned medium were used as baseline controls.	Cell viability, osteogenic differentiation markers (RUNX2, OCN, BMP-2), and activation of Wnt/TGF-β signaling were assessed.	The combination of GMSCs conditioned medium and laser irradiation significantly enhanced osteogenesis compared with controls. Upregulation of osteogenic markers and activation of Wnt/TGF-β pathway confirmed synergistic effects.
Wu, 2023 [20]	In vitro study on PDLSCs derived from extracted human teeth.	Cells irradiated with a Nd:YAG laser at sub-ablative low-energy settings (0.25–1.5 W, 30 s, MSP mode). Different power levels were tested to identify the optimal range for cell stimulation.	Non-irradiated PDLSCs served as controls.	Cell proliferation evaluated by CCK-8 assay, migration tested by Transwell assays, and gene/protein expression of SDF-1/CXCR4 signaling assessed by RT-PCR and Western blot.	Nd:YAG laser irradiation at 1 W significantly enhanced PDLSC proliferation and migration compared to controls. These effects were mediated through SDF-1/CXCR4 signaling, indicating potential for improved stem cell homing in regenerative therapy.
Talebi-Ardakani, 2016 [21]	In vitro study on primary human gingival fibroblasts cultured under standard laboratory conditions.	Fibroblasts were exposed to Er:YAG and Er,Cr:YSGG lasers at sub-ablative energy settings, applied in a controlled in vitro environment.	Non-irradiated fibroblasts were used as controls.	Cell proliferation and viability were assessed by MTT assays.	Both Er:YAG and Er,Cr:YSGG lasers increased fibroblast proliferation compared to controls, suggesting potential for enhanced soft tissue healing in periodontal therapy.

Abbreviations: ALP (Alkaline Phosphatase), BMP-2 (Bone Morphogenetic Protein-2), CCK-8 (Cell Counting Kit-8), CXCR4 (chemokine receptor type 4), Er,Cr:YSGG (Erbium, Chromium:YSGG laser), Er:YAG (Erbium:YAG laser), ERK (Extracellular Signal-Regulated Kinase), GMSCs (Gingival Mesenchymal Stem Cells), LED (Light-Emitting Diode), LLLT (Low-Level Laser Therapy), MTT (3-(4,5-dimethylthiazol-2-yl)-2,5 diphenyltetrazolium bromide assay), Nd-YAG (Neodymium:YAG laser), (OCN (Osteocalcin), PBM (Photobiomodulation), PDLSCs (Periodontal Ligament Stem Cells), RUNX2 (Runt-Related Transcription Factor 2), RT-PCR (Reverse Transcription Polymerase Chain Reaction), SDF-1 (Stromal Cell-Derived Factor-1), TGF-β (Transforming Growth Factor-Beta), Wnt (Wingless/Integrated Signaling Pathway).

**Table 4:** Ex vivo studies on laser applications for periodontal regeneration.

Author, Year	Study Design & Sample	Intervention	Comparator	Outcomes	Key Findings
Kalaivani, 2025 [22]	Ex vivo study on platelet concentrates (A-PRF and A-PRF+) prepared from human venous blood samples.	Diode laser irradiation at 630 nm applied in a non-contact mode for 15–20 s to stimulate growth factor release.	PRF and A-PRF samples not exposed to laser irradiation served as controls.	Quantification of PDGF-BB release using ELISA assays.	Laser irradiation significantly increased PDGF-BB release compared with non-irradiated controls, indicating that PBM enhances the regenerative potential of platelet concentrates.
Satish, 2023 [23]	Ex vivo study on periodontally compromised root surfaces collected from extracted human teeth.	Er,Cr:YSGG laser irradiation applied to root surfaces for smear layer removal and surface conditioning.	Root surfaces treated with EDTA or tetracycline, as well as untreated roots, were used as comparators.	Evaluation of smear layer removal and fibrin adhesion by SEM and histological analysis.	Laser irradiation effectively removed the smear layer and promoted fibrin adhesion, producing root surfaces more favorable for periodontal regeneration compared to conventional chemical conditioning methods.

Abbreviations: A-PRF (Advanced Platelet-Rich Fibrin), A-PRF+ (Advanced Platelet-Rich Fibrin Plus), ELISA (Enzyme-Linked Immunosorbent Assay), Er,Cr:YSGG (Erbium, Chromium:YSGG laser), PDGF-BB (Platelet-Derived Growth Factor-BB), PRF (Platelet-Rich Fibrin), SEM (Scanning Electron Microscopy).

**Table 5:** In vivo study on laser applications for periodontal regeneration.

Author, Year	Study Design & Model	Intervention	Comparator	Outcomes	Key Findings
Takemura, 2024 [24]	Animal study conducted on a rat model with surgically created periodontal defects.	Periodontal defects irradiated with Er:YAG laser in LLLT mode at sub-ablative parameters, repeated over the healing period.	Sham-irradiated defects served as controls.	Histological assessment of tissue repair, VEGF expression, angiogenesis, and new bone formation.	Er:YAG laser irradiation promoted angiogenesis and VEGF expression, leading to significantly greater new bone formation compared with controls, confirming its regenerative potential in vivo.

Abbreviations: Er:YAG (Erbium-doped Yttrium Aluminum Garnet), LLLT (Low-Level Laser Therapy), VEGF (Vascular Endothelial Growth Factor).

**Table 6:** Clinical studies on laser applications for periodontal regeneration: Case reports and RCTs included.

Author, Year	Study Design & Sample	Intervention	Comparator	Follow-Up	Outcomes	Key Findings
Bhardwaj, 2016 [28]	Case report on a single patient presenting with an intraosseous periodontal defect managed with regenerative surgery.	Treatment consisted of DBM graft combined with adjunctive LLLT using an 810 nm diode laser in PBM mode, applied post-surgically to stimulate healing.	No direct comparator; results were evaluated against conventional outcomes reported in the literature.	12 months.	Clinical parameters (PD reduction, CAL gain) and radiographic bone fill.	The case showed a marked reduction in PD, significant CAL gain, and radiographic evidence of bone regeneration, supporting LLLT as an adjunct to grafting.
Cetiner, 2024 [25]	RCT including 40 intrabony defect sites in patients with chronic periodontitis.	Adjunctive diode laser at 970 nm for aPDT combined with LED PBM, applied alongside GTR with biomaterials.	Control group received GTR with biomaterials but no laser therapy.	12 months.	PD, CAL, biochemical markers of bone metabolism.	Adjunctive laser therapy resulted in greater PD and CAL improvements and increased bone marker levels compared with the control group.
Dadas, 2025 [26]	RCT involving 45 patients with periodontitis undergoing SRP.	LANAP protocol with Nd:YAG laser combined with adjunctive LLLT applied after SRP.	SRP alone served as control.	12 months.	PD, CAL, radiographic bone regeneration.	LANAP + LLLT produced superior clinical and radiographic outcomes compared with SRP alone, confirming enhanced regenerative effects.
Deepthi, 2024 [29]	Case report of one patient requiring preservation of a second molar affected by periodontitis.	Combined use of diode laser at 970 nm and PBM with adjunctive PRF.	Standard care approaches reported in the literature served as comparison.	6 months.	PD, CAL, radiographic bone fill.	Laser combined with PRF reduced PD, improved CAL, and demonstrated radiographic regeneration, supporting tooth preservation.
Psg, 2025 [27]	RCT including 32 patients with intrabony periodontal defects.	Adjunctive LLLT using a diode laser during simplified papilla preservation flap surgery.	Control group underwent papilla preservation flap surgery without LLLT.	6 months.	PD, CAL, molecular markers (RUNX2, BMP-2, COL1, OPN).	LLLT enhanced clinical improvements and upregulated osteogenic biomarkers, confirming both clinical and molecular regenerative benefits.
Puthalath, 2023 [30]	Case report of a patient with stage IV periodontitis requiring regenerative treatment.	Diode 940 nm laser-assisted curettage (LANAP-like protocol).	Conventional curettage outcomes described in the literature served as comparator.	6 months.	PD, CAL, radiographic bone regeneration.	Laser-assisted curettage promoted periodontal healing and radiographic bone regeneration in advanced periodontitis.
Tan, 2022 [31]	Case report of two patients with periodontal bone defects treated with regenerative surgery.	Er:YAG laser applied in combination with A-PRF+.	Conventional regenerative approaches in the literature were used as comparators.	36 months.	PD, CAL, radiographic bone stability.	Laser combined with A-PRF+ provided stable long-term PD reduction, CAL gain and radiographic bone regeneration over three years.

Abbreviations: aPDT (Antimicrobial Photodynamic Therapy), A-PRF+ (Advanced Platelet-Rich Fibrin Plus), BMP-2 (Bone Morphopgenetic Protein 2), CAL (Clinical Attachment Level), COL1 (Collagen Type I), DBM (Demineralized Bone Matrix), Er:YAG (Erbium:YAG laser), GTR (Guided Tissue Regeneration); LANAP (Laser-Assisted New Attachment Procedure), LED (Light-Emitting Diode), LLLT (Low-Level Laser Therapy), Nd-YAG (Neodymium:YAG laser), OPN (Osteopontin), PBM (Photobiomodulation), PD (Probing Depth), PRF (Platelet-Rich Fibrin), RCT (Randomized Controlled Trial), RUNX2 (Runt-Related Transcription Factor 2), SRP (Scaling and Root Planing).

## 4. Discussion

This scoping review mapped the available evidence regarding the use of lasers in periodontal tissue regeneration across in vitro, ex vivo, in vivo and clinical settings. Overall, the findings indicate that laser therapy can exert beneficial biological and clinical effects, although heterogeneity in study designs, laser types and parameters limits the comparability of results.

In vitro studies consistently demonstrated that laser irradiation promotes key biological processes relevant to periodontal regeneration. Diode lasers at 970 and 980 nm enhanced proliferation and osteogenic differentiation of PDLSCs and potentiated the effects of stem cell–derived factors (SDF) [18,19]. Nd:YAG irradiation facilitated PDLSC proliferation and migration via the Stromal Cell-Derived Factor-1 (SDF-1)/chemokine receptor type 4 (CXCR4) pathway, supporting the hypothesis that this laser may stimulate endogenous stem cell homing [20]. Red LED light induced ERK 1/2 activation, osteogenic marker expression and mineral deposition [6]. Er:YAG and Er,Cr:YSGG lasers also improved gingival fibroblast proliferation [21]. Collectively, these studies indicate that different wavelengths can modulate cell proliferation, migration and differentiation, which are essential events for periodontal regeneration.

Ex vivo studies added evidence on the interaction between lasers and biological substrates relevant to regenerative therapy. Diode irradiation increased the release of Platelet-Derived Growth Factor-BB (PDGF-BB) from platelet concentrates (PRF and A-PRF), enhancing their growth factor content and regenerative potential [22]. Similarly, Er,Cr:YSGG irradiation effectively removed smear layer and improved fibrin adhesion on periodontally compromised root surfaces, creating more favorable conditions for periodontal attachment and regeneration [23]. These findings suggest that lasers can potentiate the efficacy of adjunctive regenerative materials used in clinical protocols.

The only one in vivo study confirmed the translational relevance of these in vitro and ex vivo results. Er:YAG laser irradiation in a rat model of periodontal defects enhanced angiogenesis, increased VEGF expression and promoted new bone formation compared with sham-irradiated controls [24]. These results support the role of Er:YAG PBM in accelerating tissue healing and regeneration in vivo.

Current clinical evidence is limited and heterogeneous; while some studies suggest a potential adjunctive benefit of laser therapy in regenerative periodontal procedures, these findings remain preliminary and should be interpreted with appropriate caution. Case reports demonstrated that diode lasers used in combination with bone grafts [28], platelet concentrates [29], or A-PRF [31] promoted significant clinical improvements and radiographic bone fill. Laser-assisted curettage using diode devices yielded radiographic regeneration in advanced periodontitis [30]. RCTs provided stronger evidence: adjunctive diode irradiation during GTR improved clinical and biochemical outcomes [25]; Nd:YAG-based Laser-Assisted New Attachment Procedure (LANAP) combined with LLLT enhanced CAL gain and bone regeneration compared to Scaling and Root planning (SRP) alone [26]; LLLT used during papilla preservation flap surgery improved clinical outcomes and upregulated osteogenic markers [27]. Together, these studies suggest that lasers may enhance the effectiveness of regenerative periodontal procedures.

When interpreting the clinical findings, it is essential to distinguish between the RCTs, which provide the most reliable evidence among the included studies, and case reports, which carry a substantially higher risk of bias and limited generalizability.

Although the clinical findings are generally promising, part of the available evidence comes from case reports and small RCTs. These designs provide useful preliminary information but inevitably occupy a lower level in the evidence hierarchy and offer limited support for broad clinical generalization. Their inclusion is appropriate within the scope of a scoping review; however, their methodological constraints should be kept in mind when interpreting the clinical outcomes reported.

A closer examination of the three included randomized controlled trials also revealed several methodological limitations. None of the studies implemented blinding of operators or outcome assessors, and randomization procedures were often incompletely described. Sample sizes were small, and although control groups were present, selective outcome reporting could not be excluded. These issues collectively introduce a moderate to high risk of bias, and the magnitude of the clinical improvements reported in these trials should therefore be interpreted with caution.

Several biological mechanisms may help explain some of the effects reported across the included studies. Photobiomodulation has been associated with increased mitochondrial activity, modulation of reactive oxygen species, and activation of intracellular signaling pathways such as ERK/MAPK, which may promote cell proliferation and differentiation. Animal studies have also shown increases in VEGF expression and angiogenesis following laser irradiation, suggesting a possible enhancement of vascularization during healing. In addition, erbium-based lasers may contribute to root surface modification and improved fibrin adhesion, potentially facilitating early clot stabilization. These mechanisms, however, should be interpreted with caution, as they are primarily supported by in vitro and preclinical evidence and may not consistently translate into predictable clinical outcomes given the current level of evidence.

Beyond these trial-specific considerations, the wider body of evidence presents additional limitations. Laser types, irradiation parameters, adjunctive regenerative materials, and outcome measures varied substantially among studies, which reduces cross-study comparability. Follow-up durations also differed widely, and only a minority of studies assessed long-term stability. Furthermore, clinical outcomes were often measured using heterogeneous indices and non-standardized definitions of periodontal healing, while in vitro and ex vivo models applied diverse experimental parameters, limiting the translational value of their findings.

Limitations inherent to the scoping review methodology must also be acknowledged. Scoping reviews do not include a formal risk-of-bias assessment and cannot determine treatment efficacy. Consequently, the findings of this review should be interpreted qualitatively rather than quantitatively. Publication bias cannot be excluded, as negative or inconclusive studies may be underrepresented in the literature. Finally, the heterogeneity of study designs, laser parameters, comparator groups, and outcome measures may have influenced the breadth and nature of the evidence captured.

Future research should focus on well-designed randomized controlled trials with standardized protocols, larger sample sizes, and long-term follow-up to clarify the clinical relevance of lasers in regenerative periodontal therapy. Moreover, mechanistic studies are needed to better elucidate how specific wavelengths and dosimetry influence cellular pathways, angiogenesis, and tissue remodeling. Finally, integration of laser therapy with advanced biomaterials and biologics represents a promising avenue for improving the predictability of regenerative strategies.

## 5. Conclusions

This scoping review highlights that, across in vitro, ex vivo, in vivo and clinical studies (RCTs and Case reports), laser therapy shows encouraging biological and clinical signals in the context of periodontal regeneration. Experimental evidence consistently points to beneficial effects on cellular activity, angiogenesis, and tissue healing, and several clinical investigations reported improvements in probing depth, clinical attachment levels and radiographic outcomes when lasers were used as adjuncts to regenerative procedures.

At the same time, the strength of the current clinical evidence remains limited by small sample sizes and methodological heterogeneity. Therefore, while the available data suggest that lasers may represent a promising adjunctive tool in regenerative periodontal therapy, these findings should still be interpreted with appropriate caution.

To clarify the true clinical relevance of laser applications, future studies should include well-designed randomized controlled trials with standardized protocols, adequate follow-up, and integration of mechanistic insights. Such research will be essential to determine whether the encouraging experimental and preliminary clinical findings can be translated into consistent and predictable clinical benefits.

## Figures and Tables

**Table 1:** Complete search strings used in each electronic database.

Database	Search String (Full Syntax)
PubMed	((“Periodontal Diseases”[MeSH] OR periodontitis[tiab] OR periodontal[tiab] OR “intrabony defect” [tiab] OR furcation[tiab]) AND (“Periodontal Regeneration”[MeSH] OR “tissue regeneration”[tiab] OR “guided tissue regeneration”[MeSH] OR GTR[tiab]) AND (“Lasers”[MeSH] OR laser[tiab] OR “Laser Therapy, Low-Level”[MeSH] OR photobiomodulation[tiab] OR LLLT[tiab] OR photodynamic[tiab] OR “Photochemotherapy”[MeSH] OR “Er:YAG”[tiab] OR “Er,Cr:YSGG”[tiab] OR Nd:YAG[tiab] OR diode[tiab] OR CO2[tiab] OR LANAP[tiab]))
Scopus	(TITLE-ABS-KEY (periodontitis OR periodontal OR “intrabony defect” OR furcation) AND TITLE-ABS-KEY (“periodontal regeneration” OR “tissue regeneration” OR “guided tissue regeneration” OR GTR) AND TITLE-ABS-KEY (laser OR “low level laser therapy” OR photobiomodulat OR LLLT OR photodynamic OR “Er:YAG” OR “Er,Cr:YSGG” OR “Nd:YAG” OR diode OR CO2 OR LANAP))
Web of Science	TS = (periodontitis OR periodontal OR “intrabony defect” OR furcation) AND TS = (“periodontal regeneration” OR “tissue regeneration” OR “guided tissue regeneration” OR GTR) AND TS = (laser OR “low level laser therapy” OR photobiomodulation OR LLLT OR photodynamic OR “Er:YAG” OR “Er,Cr:YSGG” OR “Nd:YAG” OR diode OR CO2 OR LANAP)

**Table 2:** Methodological appraisal of included randomized controlled trials.

Study	Randomization Reported	Blinding	Control Group Appropriate	Selective Outcome Reporting	Overall Appraisal
Cetiner, 2024 [25]	Partially described	No blinding	Yes	Unclear	Moderate risk
Dadas, 2025 [26]	Reported	No blinding	Yes	Unclear	Moderate risk
Psg, 2025 [27]	Not clearly described	No blinding	Yes	Possible	High risk

## Data Availability

No new data were created or analyzed in this study.

## Supplementary Materials

The following supporting information can be downloaded at https://www.mdpi.com/article/10.3390/medicina61122199/s1, Supplementary Table S1. PRISMA-ScR Checklist.
